# Prediction of Disease Free Survival in Laryngeal and Hypopharyngeal Cancers Using CT Perfusion and Radiomic Features: A Pilot Study

**DOI:** 10.3390/tomography7010002

**Published:** 2021-02-05

**Authors:** Sean Woolen, Apurva Virkud, Lubomir Hadjiiski, Kenny Cha, Heang-Ping Chan, Paul Swiecicki, Francis Worden, Ashok Srinivasan

**Affiliations:** 1 Department of Radiology, Michigan Medicine, University of Michigan, Ann Arbor, MI 48109, USA; woolensean@gmail.com (S.W.); avirkud@umich.edu (A.V.); lhadjisk@med.umich.edu (L.H.); Kenny.Cha@fda.hhs.gov (K.C.); chanhp@med.umich.edu (H.-P.C.); 2 Department of Medical Oncology, Michigan Medicine, University of Michigan, Ann Arbor, MI 48109, USA; pswiecic@med.umich.edu (P.S.); fworden@med.umich.edu (F.W.)

**Keywords:** laryngeal cancer, AT-101, CT perfusion, radiomics

## Abstract

(1) Purpose: The objective was to evaluate CT perfusion and radiomic features for prediction of one year disease free survival in laryngeal and hypopharyngeal cancer. (2) Method and Materials: This retrospective study included pre and post therapy CT neck studies in 36 patients with laryngeal/hypopharyngeal cancer. Tumor contouring was performed semi-autonomously by the computer and manually by two radiologists. Twenty-six radiomic features including morphological and gray-level features were extracted by an internally developed and validated computer-aided image analysis system. The five perfusion features analyzed included permeability surface area product (PS), blood flow (flow), blood volume (BV), mean transit time (MTT), and time-to-maximum (Tmax). One year persistent/recurrent disease data were obtained following the final treatment of definitive chemoradiation or after total laryngectomy. We performed a two-loop leave-one-out feature selection and linear discriminant analysis classifier with generation of receiver operating characteristic (ROC) curves and confidence intervals (CI). (3) Results: 10 patients (28%) had recurrence/persistent disease at 1 year. For prediction, the change in blood flow demonstrated a training AUC of 0.68 (CI 0.47–0.85) and testing AUC of 0.66 (CI 0.47–0.85). The best features selected were a combination of perfusion and radiomic features including *blood flow and computer-estimated percent volume changes-*training AUC of 0.68 (CI 0.5–0.85) and testing AUC of 0.69 (CI 0.5–0.85). The laryngoscopic percent change in volume was a poor predictor with a testing AUC of 0.4 (CI 0.16–0.57). (4) Conclusions: A combination of CT perfusion and radiomic features are potential predictors of one-year disease free survival in laryngeal and hypopharyngeal cancer patients.

## 1. Introduction

The larynx is an important organ for performing vital functions such as swallowing, speaking, and breathing naturally [1]. Treatment of advanced laryngeal cancer is challenging due to the desire to achieve disease free survival and preserve laryngeal function. Surgical management with total laryngectomy is an effective treatment strategy, but it comes with decreased quality of life [2]. Patients must learn to cope with losing their natural voice, changes in how they swallow, and overcome social stigma, which has been shown to lead to psychological and psychiatric problems [2,3].

During the 1990s, several larynx preservation clinical trials advanced the treatment of laryngeal cancer by demonstrating similar efficacy and improved quality of life with induction chemotherapy followed by definitive chemoradiation instead of total laryngectomy [4,5,6]. However, selecting which individuals will benefit from definitive chemoradiation instead of surgery is difficult. Typically, selection between different treatment options is performed by chemoselection [7], a strategy that determines the primary tumor’s response after induction chemotherapy; responders receive definitive chemoradiation and non-responders receive total laryngectomy [7]. However, no predictors exist in the literature to determine which patients will have persistent or recurrent disease after definitive treatment.

Given the importance of determining which tumors are prone to fail definitive treatment regimens, our group wanted to explore both structural conventional imaging features of tumors as well as their functional features such as computer tomography (CT) perfusion parameters as predictors. Hence, the objective of our pilot study was to investigate the feasibility of using radiomic and CT perfusion features as predictors to determine which patients with laryngeal or hypopharyngeal cancer will have persistent or recurrent disease one year after definitive chemoradiation or total laryngectomy.

## 2. Method and Materials

This Health Insurance Portability and Accountability Act (HIPAA)-compliant retrospective initiative was approved by the host institutional review board. No extramural funding was used.

### 2.1. Data Set

As part of an effort to study and optimize outcomes of advanced stage laryngeal cancer, we conducted a pilot study of identifying radiomic and CT perfusion features for treatment response prediction by machine learning methods. The data set was retrospectively taken from a phase II clinical trial in patients with stage 3 and 4 squamous cell carcinoma of the larynx or hypopharynx and receiving induction and concurrent chemotherapy with docetaxel, cisplatin, and oral AT101 [8]. All patients were potentially curable by conventional surgery or concurrent chemoradiation therapy, and had been consented for all aspects of the clinical trial prospectively including the CT neck scans with CT perfusion imaging.

Responders versus non-responders were determined by otolaryngologist during laryngoscopic exam after a single cycle of chemotherapy. Responders had a ≥50% decrease in size of tumor and non-responders had <50% decrease in size of tumor. Patients who did not achieve one-year disease free survival (DFS) were defined as patients with persistent disease at the end of definitive treatment, or recurrence within one year after the last treatment with chemoradiation, or underwent surgery with total laryngectomy.

The data set contained 88 CT scans from 44 patients who underwent pre- and post-induction chemotherapy contrast enhanced CT neck scans with additional CT perfusion imaging during December 2014–July 2018. Each patient received a pre-treatment CT scan and another one after the 1st cycle of induction. All 44 lesions examined were primary laryngeal (42/44, 95%) or hypopharyngeal (2/44, 5%) squamous cell carcinomas. A minority [11%, (5/44)] of patients were human papillomavirus positive. The mean age of patients was 59 ± 11 years old and almost all patients were male [88%, (39/44)]. Out of the 44 patients, there were eleven non-responders (11/44, 25%) and thirty-three responders (33/44, 75%). Out of the total number of patients, a partial data set of 36 (36/44, 81%) patients had treatment follow-up at one year and 8 (8/44, 18%) patients did not have follow-up at one year. Out of the partial data set of 36 patients, 26 patients achieved DFS (26/36, 72%) and 10 patients did not achieve DFS (10/36, 28%). Out of the 10 patients without DFS at one year, five patients had persistent disease after definitive therapy (5/36, 14%) and five had disease recurrence within one year (5/36, 14%).

### 2.2. CT Perfusion Technique

All 44 patients underwent initial limited non-contrast CT neck scans (1.25 mm slice thickness) focused on the larynx/hypopharynx followed by CT perfusion (Lightspeed Ultra; General Electric Medical Systems, Milwaukee, WI, USA) before and after induction chemotherapy. The Z axis coverage in each patient was determined by a head and neck radiologist who chose the levels based on the initial non-contrast CT scan to ensure adequate coverage of the tumor during CT perfusion acquisition. The CT perfusion technique consisted of injecting 50 mL of nonionic contrast (4 mL/s; Ultravist 300; Bayer Health Care, Wayne, NJ, USA) followed by 20 mL normal saline at 4 mL/s, and cine acquisition starting 5 s into the injection. The cine parameters were set at 120 kV and 60 mA. The cine images covered a 4 cm area, as 8 images were taken with a 5-mm slice thickness per cine rotation. Finally, contrast enhanced CT neck images were obtained from the skull base to the thoracic inlet after administration of an additional 75 mL of nonionic contrast (4 mL/s; Ultravist 300; Bayer Health Care, Wayne, NJ, USA) followed by 20 mL normal saline at 4 mL/s.

### 2.3. CT Perfusion Post Processing

**Figure 1:**
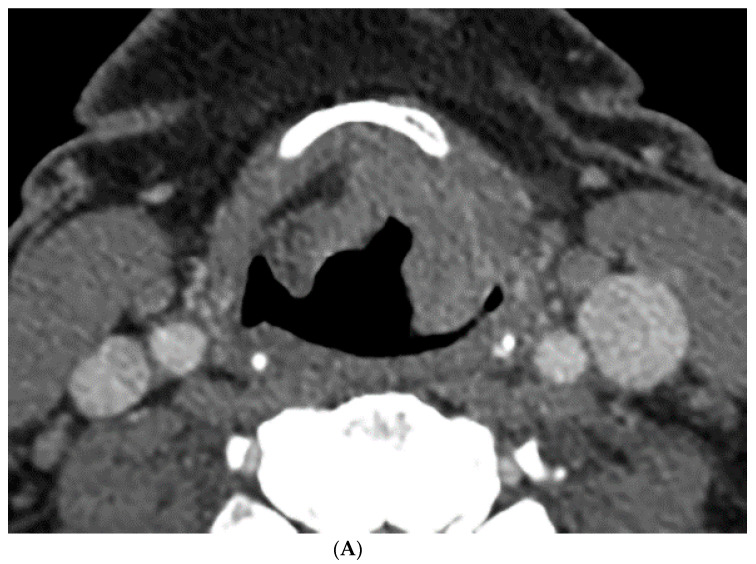

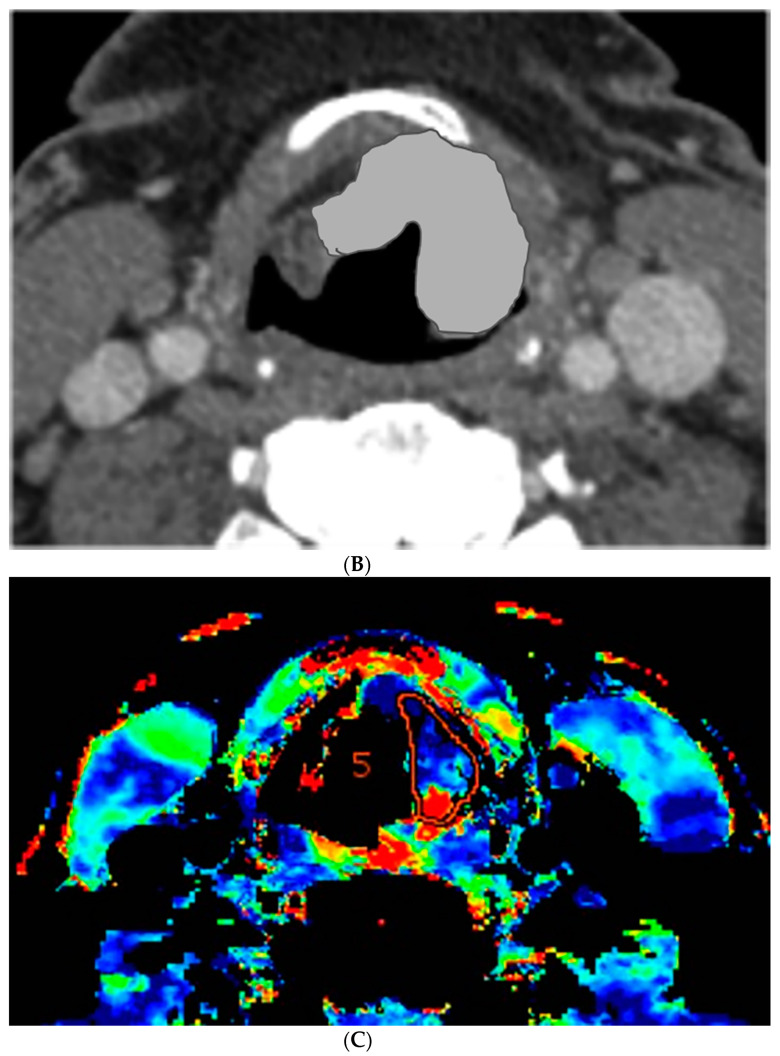
Computer tomography (CT) and perfusion sections of laryngeal carcinoma on pre-treatment images. Axial sections show the grayscale image (**A**) and outlines for the following: computer (**B**) and perfusion image (**C**). Note that the region of interest (ROI) drawn on the CT perfusion image appears different in size from that on the contrast enhanced image; this is because the CT perfusion images are 5 mm in slice thickness whereas the conventional CT images are 1.25 mm in slice thickness (implying that the CT perfusion data is a composite of 4 adjacent 1.25 mm slices from conventional CT).

**Figure 2:**
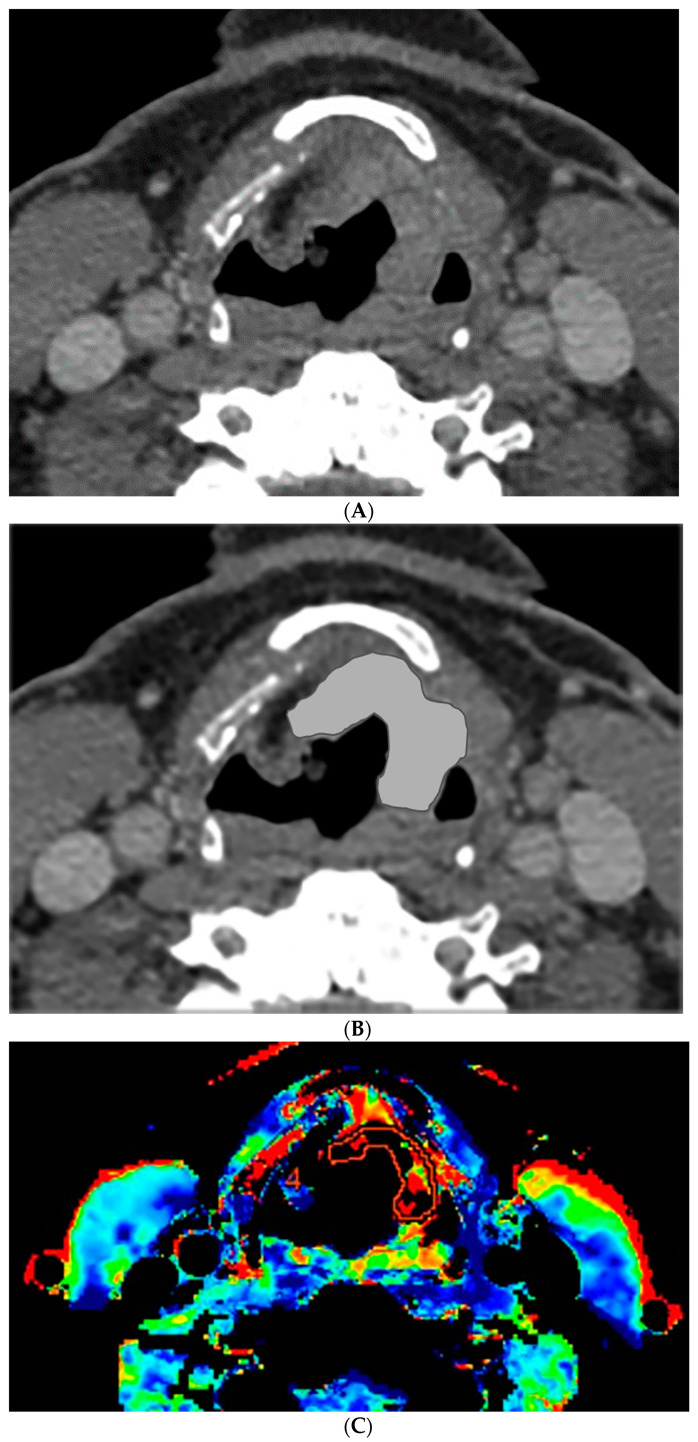
CT and perfusion sections of laryngeal carcinoma on post-treatment images. Axial sections show the grayscale image (**A**) and outlines for the following: computer (**B**) and perfusion (**C**).

The perfusion data post processing was performed on a commercially available Perfusion-4 software package on an Advantage Windows Workstation version 4.0 (General Electric Medical Systems, Milwaukee, WI, USA). An oval region of interest was placed in the internal carotid artery to generate the contrast arterial enhancement curve. Radiologist 1 (a fellowship trained neuroradiologist with 15 years of experience in head and neck imaging) drew additional freehand regions of interest contouring the tumor along its margin on all axial images where the tumor was visible, and an oval region of interest in normal muscle tissue. An example of the region of interest through the tumor is demonstrated in Figure 1 and Figure 2. The perfusion data were post processed by a deconvolution-based method into maps that represented permeability surface area product (PS), blood flow (BF), blood volume (BV), mean transit time (MTT), and time-to-maximum (Tmax).

### 2.4. Segmentation of Head and Neck Lesions

Radiologist 1 in this study is a fellowship trained neuroradiologist with 15 years of experience in head and neck imaging and Radiologist 2 is a senior radiology resident with 4 years of experience in head and neck imaging. Contouring of pretreatment and post-induction chemotherapy tumors on contrast enhanced CT neck images was performed by the two radiologists manually and semi-autonomously by the computer with input approximate bounding boxes provided by Radiologist 1. The tumors were contoured on all the consecutive axial contrast enhanced CT neck slices that contained the tumor by manually drawing the boundaries that best distinguished the margins of the tumor from normal surrounding tissue. Since tumor heterogeneity is a potential feature that influences treatment response and survival, we elected to include all portions of the tumor, both well enhancing and poorly enhancing; moreover, none of the tumors had large necrotic areas that could be isolated from the enhancing tumor. Previous research found good agreement between automated 3D segmentation by our in-house developed software tool and radiologists’ manual segmentation for assessing tumor volume change in a variety of head and neck tumors [9,10]. We applied our automated segmentation tool to the data set in this study. Briefly, the segmentation method consists of three stages including preprocessing, initial segmentation, and 3D level-set segmentation with an approximate bounding box of the tumor as input. In the first stage, smoothed images and gradient images are generated by applying 3D preprocessing techniques including smoothing, anisotropic diffusion, gradient filtering, and rank transform of the gradient magnitude. The second stage includes multiple steps to obtain an initial segmentation: first, a set of pixels in the center of the lesion is assumed to represent statistically a sample of the full population of pixels; second, a preliminary contour is obtained by thresholding the pixels falling within 3 standard deviations of the mean of the pixels in the center of the lesion and with values above -400 HU; finally, an initial segmentation is obtained by applying morphologic dilation, 3D flood filling, and morphologic erosion operations to the preliminary contour. In the third stage, the final contour is obtained by level-set segmentation utilizing both 3D and 2D level sets to refine and smooth the initial contour and pull the contour towards the tumor edges.

### 2.5. Feature Extraction

Tumor feature extraction was performed by an internally developed and validated computer-aided image analysis system [11,12,13]. A total of twenty-six radiomic features and five CT perfusion features were extracted from each tumor in the data set. The morphological and grayscale radiomic features included categories of volume, contrast and shape. The perfusion features included: PS, BF, BV, MTT, and Tmax. The clinical laryngoscopic features included the clinically reported percent treatment response after definitive therapy and the binary classification as a responder or non-responder.

### 2.6. Evaluation Methods

Interobserver variability was calculated to ensure the internal validity of the computer segmentation and radiologist as well as to evaluate the laryngoscopic correlation with the radiologist and the computer. Interclass correlations (ICC) were analyzed on pre-to-post-treatment percent volume change for the semi-autonomous computer segmentation, Radiologist 1 manual segmentation, Radiologist 2 manual segmentation, and endoscopic evaluation by the otolaryngologist. A two-sided paired t-test was performed to estimate statistical significance.

Our primary goal was to predict which tumors would not achieve DFS at one year after definitive treatment using the pre-to-post induction chemotherapy changes in the radiomic, perfusion, and clinical laryngoscopic features. We used a two-loop leave-one-out feature selection and a linear discriminant analysis classifier to merge the selected features into a combined response index as decision variable. The receiver operating characteristics analysis was performed using the ROCKIT software (Version 0.9.1-beta, CE Metz, University of Chicago, Chicago, IL, USA) and an area under the receiver operating characteristic curve (AUC) with 95% confidence intervals (CI) was calculated to estimate the performance for prediction. Additionally, a kappa analysis was performed for the binary classification of clinical laryngoscopic responder and nonresponder.

## 3. Results

### 3.1. Percent Volume Change Correlation

The percent volume change ICC of the computer with Radiologist 1 or Radiologist 2 were 0.80 and 0.74, respectively. The percent volume change ICC between the two radiologists was 0.73. The differences in the computer-versus-radiologist and radiologist-versus-radiologist ICCs did not achieve statistical significance. Examples of the semi-autonomous computerized 3D level-set segmentation are demonstrated in Figure 1 and Figure 2.

The percent volume change ICC of the endoscopic evaluation with Radiologist 1 or Radiologist 2 were 0.49 and 0.58, respectively. The percent volume change ICC of the endoscopic evaluation with the computer was 0.56. None of the differences between pairs of these ICCs achieved statistical significance. The variation in percent volume change estimates resulted in change in management classification in 13 cases (13/36, 36%) by the computer, 7 of which (7/36, 19%) by both the computer and the radiologists.

### 3.2. Disease Free Survival

**Figure 3:**
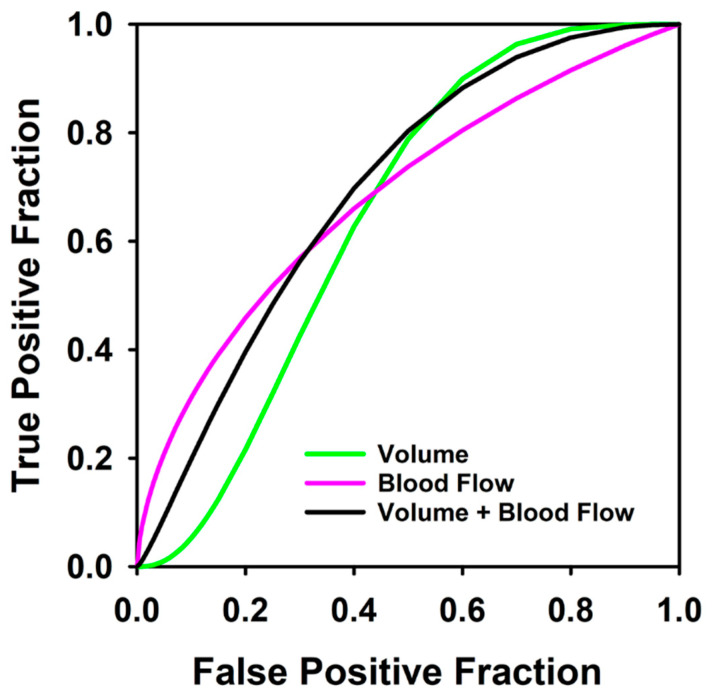
Receiver operating characteristic curves for predicting which patients would not achieve disease free survival at 1 year using blood flow change alone (AUC = 0.66), percent volume change by computer contouring (volume) (AUC = 0.64), and the combined response index for 36 pre- and post-induction therapy tumors (AUC = 0.69).

**Figure 4:**
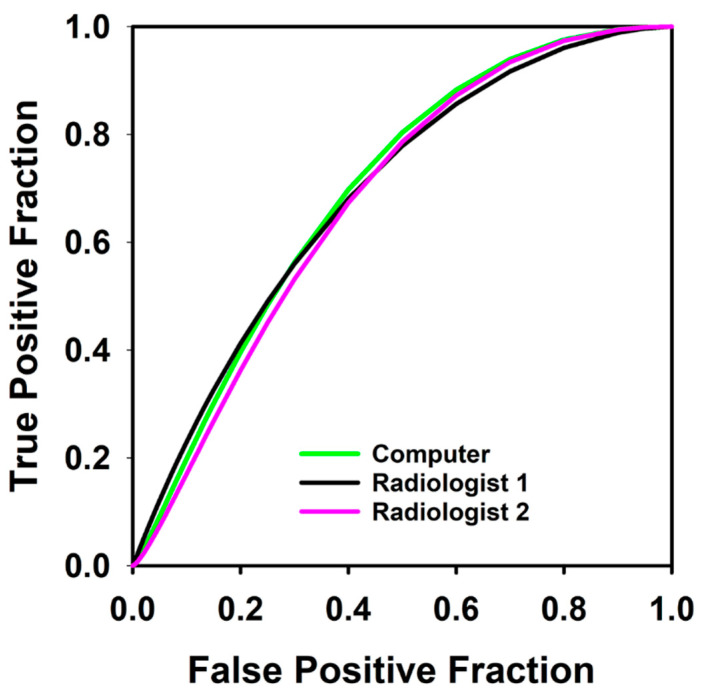
Receiver operating characteristic curves for predicting which patients would not achieve disease free survival at 1 year using combined response index of blood flow and percent volume change calculated from contours by computer, Radiologist 1, and Radiologist 2 in 36 pre- and post-induction therapy tumors. AUC for the computer, Radiologist 1 and Radiologist 2 were 0.69, 0.71 and 0.68 respectively.

The AUCs for our primary experiment predicting which patients would not achieve DFS after definitive treatment are shown in Table 1, and the ROC curves are shown in Figure 3 and Figure 4. The best feature was the *change in blood flow* with a training AUC of 0.68 (CI 0.47–0.85) and testing AUC of 0.66 (CI 0.47–0.85). The computer’s combined response index using both radiomic and perfusion features of change in volume and change in blood flow improved slightly to a training AUC of 0.68 (CI 0.5–0.85) and testing AUC of 0.69 (CI 0.5–0.85). None of the differences between pairs of these AUCs achieved statistical significance.

The clinical laryngoscopic features were not good predictors for which patients would not achieve one-year DFS. An experiment using the laryngoscopic treatment response after induction chemotherapy as predictor had a training AUC of 0.33 (CI 0.16–0.57) and testing AUC of 0.4 (CI 0.16–0.57). The differences between the AUCs did not achieve statistical significance. An experiment using the clinical laryngoscopic classification of responder and non-responder as binary outcomes showed poor correlation with the response at one year after definitive chemoradiation with a kappa value of less than chance (k = −0.01).

## 4. Discussion

Selection of the appropriate management with definitive chemoradiation or total laryngectomy is an important phase in the treatment of advanced laryngeal cancer. Treatment of advanced laryngeal cancer without the appropriate classification results in decreased quality of life if an unnecessary total laryngectomy is performed or increased morbidity with prolonged therapy if definitive chemoradiation is performed on a nonresponder. Our pilot study suggests that CT perfusion and radiomic features may be used as predictors for determining which patients would not achieve disease free survival. The combined response index that used the features of change in blood flow and change in volume after induction chemotherapy achieved a testing AUC of 0.68 for predicting which patients would not achieve one-year DFS. Even though this AUC result is modest, it still outperformed the current clinical laryngoscopic evaluation in our pilot data set. This suggests that computer-aided image analysis has the potential to provide additional predictive information to supplement otolaryngologist decision making for performing organ preserving therapy versus surgery.

In our study, the percent volume change after induction chemotherapy with laryngoscopic evaluation was a poor predictor for the small subset of tumors that had persistent or recurrent disease with an AUC of 0.4. The best combined response index used both CT perfusion and radiomic features with a testing AUC of 0.68. Thus, our pilot study suggests that treatment selection with laryngoscopic evaluation after induction chemotherapy may not correctly identify the subset of patients that would have persistent or recurrent disease. The ability of CT perfusion imaging to predict the ‘difficult to treat’ tumors shows the potential to add value in the treatment selection process and counseling of the patient. However, the data set was small in our pilot study and follow-up studies with larger patient cohort are needed to confirm the trend.

The computer ICC between the radiologist and clinical endoscopic results did not demonstrate a significant difference compared to ICC between the radiologists. However, the difference in the percent volume changes estimated from segmentation by the computer or the radiologists, and by laryngoscopic evaluation resulted in a change of non-responder and responder classification in 13 cases (13/36, 36%) by the computer and 7 cases (7/36, 19%) with both the computer and the radiologists in agreement using a similar 50% change in volume threshold after induction chemotherapy. Therefore, the measurement variation in percent volume change between measurement methods can result in different treatment response classifications. A 3D segmentation of laryngeal tumors by the computer guided by the radiologist with a percent volume change calculation would likely be a desirable service to the otolaryngologist. Such data could be used by the otolaryngologist to supplement their laryngoscopic evaluation and potentially minimize the variation in the percent volume change assessments.

Prior literature has shown that CT perfusion features are promising for assessing treatment response with chemoradiation [14,15]. A study prospectively conducted by Rana et al. [14] evaluated neck CT perfusion characteristics in 24 patients and found that blood flow alone was 83.3% predictive of response to chemoradiation irrespective of stage. Truong et al. [15] analyzed the serial changes in head and neck tumor perfusion in 15 patients and found increased pre-treatment tumor blood flow and increased blood flow from pre-treatment scan two weeks after radiation therapy in patients that achieved locoregional response. However, none of these studies evaluated the ability of perfusion features to predict which tumors will not achieve DFS at one year after definitive therapy.

Our work has limitations. Since this is a pilot study, the data set is small which can introduce bias even with robust computer-aided image analysis techniques. In a future study, the data set will be enlarged to assess the generalizability of the predictors. Inter-observer and intra-observer variability in percent volume change measurements exist between the two radiologists, semi-autonomous computer segmentation, and endoscopic evaluation. However, the differences did not achieve statistical significance between the ICCs of the computer with the radiologist or laryngoscopic evaluation and the ICC of Radiologist 1 with Radiologist 2.

In conclusion, our pilot study indicates that CT perfusion BF feature is a promising predictor for assessing which tumors will not achieve one-year DFS. The addition of radiomic features minimally trend towards increasing the predictive ability of BF. This study indicates that radiologist-guided computer-aided prediction of tumor recurrence and persistent disease has the potential to help oncologist and otolaryngologist provide a more informed counseling session to patients deciding between laryngeal preserving therapy or total laryngectomy.

## Figures and Tables

**Table 1:** Training and testing area under the receiver operating characteristic (ROC) curve (AUC) with 95% confidence intervals (CI) for predicting which patients would not achieve disease free survival at 1 year using change in blood flow (BF) + percent change in volume (volume) for 36 pre- and post-induction therapy tumors. BF was estimated within Radiologist 1′s defined regions by vendor’s software, percent volume change was calculated with computer or Radiologist 1 or Radiologist 2′s manual segmented contours as labeled in column 1.

Features	BF AUC (CI)	Volume AUC (CI)	BF + Volume AUC (CI)
**Computer** **Training**	x	x	0.68 (0.50, 0.85)
**Computer** **Testing**	x	0.64 (0.46, 0.80)	0.69 (0.50, 0.85)
**Radiologist 1** **Training**	0.68 (0.47, 0.85)	0.52 (0.29, 0.73)	0.69 (0.49, 0.85)
**Radiologist 1** **Testing**	0.66 (0.47,0.85)	0.58 (0.29, 0.73)	0.71 (0.49,0.85)
**Radiologist 2** **Training**	x	0.53 (0.37, 0.74)	0.68 (0.48, 0.84)
**Radiologist 2** **Testing**	x	0.58 (0.37, 0.74)	0.68 (0.48, 0.84)

## Data Availability

Data available on request due to HIPAA restrictions eg privacy or ethical.
